# Retrospective duration judgments of naturalistic events depend on memories of event boundaries

**DOI:** 10.3758/s13423-025-02833-z

**Published:** 2026-01-05

**Authors:** Winny W. Y. Yue, Jing Liu, Ziqing Yao, Yuqi Zhang, Zexuan Mu, Xiaoqing Hu

**Affiliations:** 1 Department of Psychology, The State Key Laboratory of Brain and Cognitive Sciences, The University of Hong Kong, Pokfulam, Hong Kong SAR, China; 2 School of Psychology, South China Normal University, Guangzhou, China; 3 HKU-Shenzhen Institute of Research and Innovation, Shenzhen, China

**Keywords:** Retrospective duration judgments, Naturalistic events, Event boundaries, Memory hierarchy

## Abstract

Daily planning and goal-directed behavior rely on accurate judgments of the duration of past experience. However, retrospective duration judgments are often inaccurate. At the same time, our memory of these experiences transforms over time, with memory forgetting being a common occurrence. In this case, whether and how changes in episodic memory impact duration judgments? Here, participants watched videos depicting daily events with clear boundaries segmenting each subevent. Participants then completed recall and duration judgment tasks both immediately and after 7 days. For whole events, results showed that the recall of the event structure, specifically the number of subevents, significantly influenced immediate and delayed duration judgments. In contrast, event content memories, including gist and recalled details, had no major impact on the entire event duration. In contrast, duration judgments of individual subevents depend on the recall of event content, with immediate judgments linked to recalled gist accuracy and detail richness, while delayed judgments tend to average out, with no significant effect from change in recalled details. Together, these results suggest that retrospective duration judgments rely on explicit episodic memory recall, with the type of recall varying depending on the size and complexity of the naturalistic event. While the segmented structure provides a consistent basis for duration judgments of complex events, single subevents without internal boundaries rely more on granular details.

**Supplementary Information:**

The online version contains supplementary material available at 10.3758/s13423-025-02833-z.

## Introduction

People can perceive event durations ranging from milliseconds to years (Karmarkar & Buonomano, 2007). Judgment of event duration can be conducted retrospectively, either immediately following an experience or after a considerable lapse of time (Grondin et al., 2014; MacDonald, 2014; Ornstein, 1969; Tsao et al., 2022; Yarmey & Matthys, 1990; Zakay & Block, 2004; Zakay & Fallach, 1984). Our ability to assess past event durations is crucial for various cognitive processes, including motor learning, strategic planning, and speech interpretation (Gransier et al., 2023; Little et al., 2013; Paton & Buonomano, 2018; Zion Golumbic et al., 2012). Despite its importance, our retrospective duration judgment is not always accurate; Research shows mixed patterns with some studies reporting overestimation of event duration (Yarmey, 2000) while others find underestimation (Hicks et al., 1976; Zakay & Block, 2004). Concurrent with these inconsistencies in duration judgment, our memories of past experiences also undergo significant changes, such as forgetting of specific details and schematic organization, leading to generalization of memory representations (Radvansky et al., 2022; Santoro et al., 2016; van Kesteren et al., 2012). However, how retrospective duration judgments are derived from our recall of specific episodic memory remains elusive.

An interesting observation is that richer recall of event content is often associated with less compressed and more accurate duration judgement (Block, 1992; Clewett et al., 2019; D’Argembeau et al., 2022; Jeunehomme et al., 2018; Jeunehomme & D’Argembeau, 2019; Zakay & Block, 2004). The early storage-size hypothesis attributed this to the quantity of retrievable memory content (Ornstein, 1969). However, subsequent research revealed that event boundaries created by contextual changes, rather than mere memory volume, predominantly determine retrospective temporal perception (Block et al., 2010; Block & Reed, 1978). In fact, these contextual boundaries shape memory organization; information within boundaries is integrated into single coherent units during encoding, forming subevents (Ben-Yakov et al., 2013; Dudai et al., 2015; Horner et al., 2016; Sun et al., 2020; Terada et al., 2017; Wallenstein et al., 1998). Importantly, compared to items within the same subevent, those that span event boundaries are perceived as more temporally distant, even if they are of the same actual distance (DuBrow & Davachi, 2016; Pu et al., 2022). Consistently, neural activation patterns during retrieval also show greater similarity for items within the same subevent but decreased similarity across boundaries (Ezzyat & Davachi, 2014; Lositsky et al., 2016). As such, event boundaries are suggested to serve as access points from which memories are retrieved, whereas granular details would be skipped (Michelmann et al., 2023).

While multiple studies have consistently found a positive relationship between retrospective perceived duration and the number of subevents recalled (Block, 1992; Block & Reed, 1978; Faber & Gennari, 2015; Jeunehomme & D’Argembeau, 2019; Poynter, 1983; Zakay et al., 1994), only one has delved deeper into examining the different role of event structure and details in retrospective duration judgment. By asking subjects to perform campus walks as naturalistic events, the authors found that subevents predicted duration judgments while granular details had only little impact (Jeunehomme & D’Argembeau, 2019). However, participants in this study were required to perform vivid mental reexperience before duration recall, which encouraged the recall of all kinds of granular details, including personal thoughts, possibly leading to further segmentation of the experience and fundamentally altering the natural processes of duration judgment. Additionally, most previous studies used static stimuli rather than continuous events, with boundaries preset or identified by external coders rather than self-detected during encoding. Most importantly, duration judgments were measured only at one point in time, so the time-dependent shifts in memory and perceived duration were not captured. This leaves open the question of whether, as time evolves and memory changes, duration judgments follow shifts in memory for event boundaries, but not for granular details within subevent.

To examine memory-related changes in duration judgments, we designed a within-subject test–retest study with a 7-day interval during which forgetting would occur, naturally altering memories of event structure and details. This approach isolate memory effects within individuals compared to between-subject designs with delayed measures, which assesses retrospective duration only once, making it difficult to determine whether differences in duration judgments stem from memory content or other variables during encoding such as attention or cognitive load (Grondin et al., 2014; Tsao et al., 2022; Yarmey & Matthys, 1990). To examine the role of event structure and details, we used videos demonstrating clear structure with distinct subevents separated by event boundaries following the event segmentation theory (Baldassano et al., 2018; Zacks & Swallow, 2007; Zacks & Tversky, 2001). We hypothesized that changes in duration judgments would differ by the recall of event structure as quantified by the number of subevents recalled, but not event content, including gist (i.e., core activities) and contextual details (e.g., objects, characters).

## Method

### Subjects

Fifty-five individuals (women = 39, preferred not to say = 1; *M*_age_ = 22.589, *SD*_age_ = 3.109) were recruited from the University of Hong Kong for this experiment. Six participants were excluded due to absenteeism in the delayed experiment, and one was excluded for low performance during the filtering process (see Analysis section), yielding a final sample size of 48 participants. We analyzed the data using a Linear Mixed Model (LMM) to examine how changes in event structure and content memory over a 7-day delay influenced retrospective duration judgments in our repeated measures design. To justify our sample size, we conducted a sensitivity power analysis using G*Power 3.1 (Faul et al., 2007). Approximating with a paired *t* test framework (a conservative estimate), the minimal detectable effect size for 48 participants is Cohen’s *d* = 0.413, with 80% power at an alpha of.05 (two-tailed). The LMM, by accounting for potential within-subject correlation, likely provides similar or greater power.

All eligible participants had normal or corrected-to-normal vision and hearing, were not color-blind, and had no chronic medical conditions, history of severe mental illness, neurological disorders, or current clinical diagnosis for any psychiatric conditions. The experiments were conducted in either simplified or traditional Chinese, with all instructions and experimental materials remaining consistent across both language versions. Participants were assigned to the language condition based on their preferred language. All participants provided written informed consent and received monetary compensation upon completing the entire experiment ($250 HKD, approximately $32 USD). This study has been approved by and conforms to the standards of the Human Research Ethics Committee for Non-Clinical Faculties (Ethnics approval number is not included in this submission for anonymization purposes).

### Materials

**Fig. 1:**
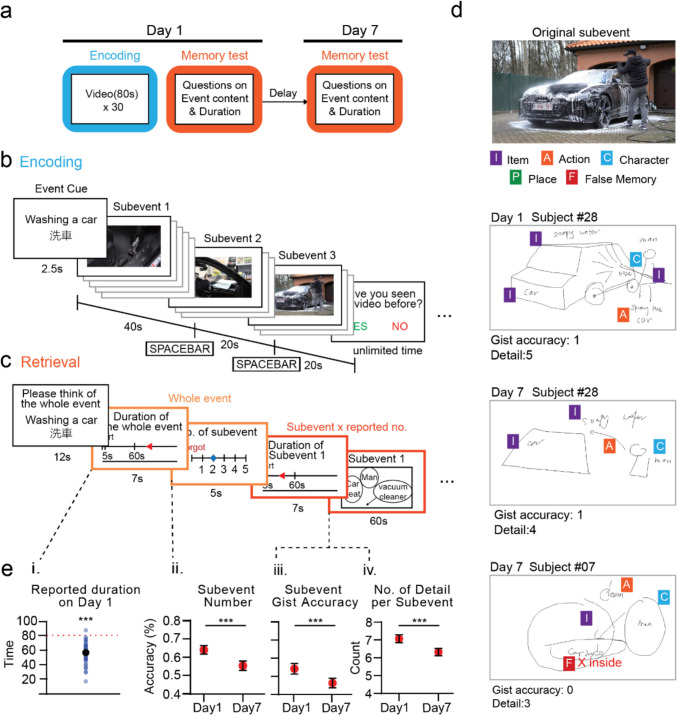
Experimental paradigm. **a** Experimental flow. **b** The encoding phase. Participants viewed thirty 80-s videos depicting daily life events, each comprising 2–4 nested subevents each lasting either 20 or 40 s. A short phrase describing the event was presented before each video as a cue. During video watching, participants were instructed to press the space bar to indicate event boundaries when context or activity shifts. **c** The retrieval phase. Prompted by cues, participants were asked to recall the corresponding video, followed by questions about the entire event and each subevent. Specifically, participants reported the duration of the whole event and of each subevent, reported the numbers of subevent in each individual event, and drew each subevent out with labels to show the remembered contents. **d** Example coding for drawings by participants. Coders first assessed whether the drawings captured the gist of the subevent, then categorized and counted the number of details the participants included. **e** (i) Participants’ duration judgments on Day 1 showed temporal compression. Blue dots represent the mean reported duration for each participant. Black dots indicate the mean reported duration for all participants. Red dotted line indicates actual event duration. (ii–iv) Changes in memory attributes across days. (ii) Reported subevent number. (iii) Recalled gist accuracy (iv) Number of details. Participants’ recall of events significantly declined from Day 1 to Day 7 (i.e., forgetting; *n* = 48). The dataset excluded invalid responses and empty responses (see Methods). See Fig. S2 for plots with memory performance before filtering. Error bars show the SEM. ***p <.001 (all two-tailed). (Color figure online)

Thirty short videos were included in the study, each uniformly lasting 80 s, for the formal experiment. We used a fixed duration to ensure experimental consistency and cross-stimulus comparability, thereby enabling examination of the effect of event boundary and memory content without the confounding influence of duration variability. These videos depict various everyday activities, for example washing a car and playing at the beach. Each video contained two to four distinct and meaningful segments (i.e., subevents) extracted from a longer raw footage (e.g., ‘vacuuming the seats’, ‘cleaning the window’, ‘hosing off the car’ in a car-washing video; see Fig. 1b). Subevent selection followed three criteria: (1) activities had to be readily distinguishable within the time constraints; (2) segments had to capture distinct nonoverlapping activities; and (3) segments had to include recognizable actions and objects describable without technical expertise. To prevent participants from relying on fixed subevent lengths to calculate total duration during the retrieval test, we adopted two subevent durations, 20 and 40 s, while still ensuring ample time for participants to fully perceive each activity. These segments were cut and combined at natural transition points (avoiding mid-activity cuts) to form structured videos with distinct boundaries. As a result, all videos have clear event structure with distinct boundaries, aligning with the coarse-grained event segmentation in a hierarchical event (Zacks & Swallow, 2007; Zacks & Tversky, 2001).

These videos are sourced from platforms like YouTube and Bilibili (a complete video list is provided in Table S6). The unselected portions of the original footage were excluded, but no frames were omitted within the chosen segments to maintain the natural flow of each activity. To maintain consistency and minimize external distractions, each subevent within an event was filmed in a single continuous shot, with no manual editing, visual effects, or subtitles. Additionally, any logos in the videos were blurred to avoid biasing participants’ perceptions. All videos were edited in Adobe After Effect and were exported with Adobe Media Encoder to resolution of 1,920 (w) × 1,080 (h).

These videos were tested in a pilot study involving three coders who assessed event boundary consistency. In this pilot study, coders viewed the edited videos and indicated event boundaries by pressing a key whenever they perceived a major event change, mirroring the segmentation task participants would perform in the main experiment. Only videos for which all three coders pressed a key within 5 s after the defined boundary were selected. This preliminary testing was conducted to minimize variations in the perception of event boundaries during the actual experiment. To familiarize participants with the procedure, four of these videos were used in a practice session. The remaining 30 videos were presented during the encoding session of the task. During the study, we replaced two videos, numbered 9 and 18, due to ambiguous event boundary identification observed in the initial experimental phase. Consequently, the trials associated with these videos from the first eight participants were excluded from the final analysis to ensure the reliability and consistency of the data.

The experiment was conducted using the software PsychoPy (Version 2022.2.4). During the retrieval test, participants were instructed to use a drawing pad (Wacom Intuos CTH-680) to complete the drawing and labeling tasks.

### Procedures

To investigate the role of memory in event duration judgment, we conducted a within-subject experiment over 7 days, tracking how alterations in memory at different structural levels influence participants’ duration assessments. Memory tests were administered immediately after participants watched the videos on Day 1 and again on Day 7 (see Fig. 1a). During these tests, participants described their memories in the form of drawings and reported the duration of each event and subevent (see Figs. 1b, c). We then systematically coded their memories and analyzed both event structure (i.e., the number of subevents) and event content, including gist as well as the quantity and categories of details for each recalled subevent (see Fig. 1d).

Given that our study primarily focuses on assessing retrospective duration judgments, participants were instructed to remove all wearable watches and turn off their electronic devices before the experiment began. To eliminate any potential time cues, all clocks in the lab and on the computer interface were removed. Any questions regarding the time were not answered to maintain the integrity of duration judgments. Participants were informed at the time of sign-up that the experiment would conclude on schedule, lasting approximately 2.5 hours for Day 1 and 1.5 hours for Day 7. Additionally, they were not informed of the number of tasks or the duration of each task before and during the experiment.

### Encoding session

During the encoding session, participants watched 30 videos depicting distinct daily life events. Before the task began, they were briefed on the structure of the events, with subevents defined as distinct segments representing different activities. Participants were informed that each event would consist of a varying number of subevents. They were instructed to engage with the videos attentively, as if personally experiencing the events. However, since the study focused on naturalistic recall, participants were neither directed to memorize the videos nor informed of an upcoming memory retrieval task.

To assess participants’ ability to recognize the transition between subevents and thereby confirm their understanding of the event structure, they were instructed to press the SPACE BAR as soon as they recognized a change in subevents while watching the videos. After each video, participants were asked whether they had seen the video before this experiment.

Before each video, a cue word was presented for 2.5 s to serve as a prompt for recall during the subsequent retrieval task. This cue word encapsulated the overall event, such as ‘washing car,’ without specifying any subevents.

### Day 1 retrieval test

Immediately following the encoding session, participants underwent a surprise test designed to structurally assess their baseline memory and duration judgments for each event and subevent. All 30 videos from the encoding session were included in this test. Each trial consisted of three parts: first, participants estimated the duration of the entire event. Next, they identified the number of subevents within the video. Finally, they provided the duration and described the content of each subevent.

In the first part of the test, participants were asked to report the total duration (in seconds) of the entire video using a slider. Since participants performed the same memory test on Day 7, we designed the slider with only minimal indicators to encourage memory-based duration judgments rather than recalling specific numerical values, e.g., “13” s. Nevertheless, to still provide sufficient orientation for making duration judgments, we included two reference points: 5 s (for second-range estimates) and 60 s (for minute-range estimates) on a scale ranging from 0 to 300 s. Without specific numerical markers, differences in duration estimates between sessions were more likely to reflect memory changes rather than remembering specific slider positions from their Day 1 session. This was particularly important for our repeated-measures approach, where we needed to isolate memory effects from other potential influences like recall bias. In the instructions, we clarified that these reference points provide indicators of duration but had no relationship to the actual video duration, and participants could select any position on the continuous slider based on their duration estimates.

Previous research allowed participants unlimited time to mentally re-experience events in detail and reported duration (Jeunehomme & D’Argembeau, 2019). While this study did not directly examine the correlation between mental re-experience time and the length of duration judgment, they reported that longer re-experience durations were associated with increased detail recall, and that greater detail recall, in turn, correlated with longer reported durations. To ensure a uniform delay between memory retrieval and duration judgment and to control for potential confounding effects stemming from varied mental rehearsal strategies, our study implemented fixed time windows: 12 s for mental recall, followed by 7 s for making temporal judgment and submitting duration estimate of the entire video. During this phase, participants were instructed to focus solely on the current task and not to consider other videos or forthcoming questions.

Next, to evaluate event structure memory, participants reported the number of subevents they recalled from the video. Participants were instructed to honestly indicate the number of subevents they could remember, even if they had forgotten some content or details, using a slider ranging from 1 to 5.

In order to investigate the relationship between memory content and temporal judgments, participants were instructed to estimate the duration and depict the content of each subevent. They provided the duration using the same slider as for the total event duration. Given the study used purely visual stimuli (i.e., videos without sounds), we asked participants to draw as the retrieval method. Participants depicted objects, characters, and background details on a blank page, based on their recollections, and were informed that clear labeling with circles could be used instead of detailed drawings. To convey the actions of the characters, participants were instructed to use arrows and labels. In fact, the subevents were carefully designed to capture single activities with minimal sub-actions and limited within-subevent contextual changes, further facilitating their representation in a static format.

We recognize the potential influence of the drawing on memory consolidation (Fernandes et al., 2018; Wammes et al., 2016, 2019). While drawing may have enhanced memory encoding, similar consolidation effects can also occur with other forms of recall, such as verbal or written recall (Krishnan et al., 2017). Participants had one minute to complete each subevent before moving on to the next. To ensure understanding and accuracy, they practiced using the drawing pad under the experimenter’s guidance during a demonstration and practice session prior to the actual task.

Throughout the task, participants who were uncertain of their responses or had minimal recall were permitted to skip the specific questions they were unsure about. Trials were fully randomized, and an untimed break was provided after every five videos. The entire task lasted approximately 1 to 1.5 hours. At the end of the experiment, participants were free to leave.

### Day 7 retrieval test

To analyze changes in memory and reported durations, participants repeated the memory test after a 7-day delay, allowing us to measure the impact of natural memory changes. This 7-day interval was determined through a pilot test to ensure sufficient trials with natural forgetting. Trials were fully randomized, and all videos from the initial test were included in this follow-up assessment.

### Data analysis

#### Exclusion criteria

One participant (Participant no. 1010) was excluded for indicating forgetting on 26/30 trials during the Day 7 retrieval test. To ensure data integrity and allow for fairer comparisons across participants, trials were excluded if participants had previously been exposed to the video before the experiment or pressed the SPACE BAR an incorrect number of times relative to the actual number of subevents in the video during movie viewing. These exclusions prevented prior knowledge from biasing the results and ensured that each participant’s initial interpretation of the event structure was aligned with the intended segmentation.

To prevent random guessing, we allowed participants to skip trials during the test session. To accurately align event content memories with duration judgments, the following trials were excluded (presented in the order they occurred during the test): (1) trials where participants indicated they had completely forgotten everything about the whole event, (2) trials with no response within the time limit or a skip (indicating the participant was unable to judge the duration) during the total duration report, and (3) trials where participants responded with “I forgot” or did not provide an answer within the time limit during the subevent number report. Additionally, to ensure the validity of responses, the analysis excluded: (1) trials exhibiting unusual temporal compression of less than 10 s for total duration judgment, (2) trials where any single subevent duration exceeded the total duration, indicating possible data entry errors. Excluding these trials ensured a more precise and reliable analysis of the remaining data (see Table S1 for the number of trial attrition).

#### The analysis of drawings

To assess participants’ recall accuracy and the details recalled, we invited coders to evaluate participants’ drawings based on gist and details. In this study, ‘gist’ refers to the main activity involved that captures what has happened in the subevent, while ‘details’ refers to the contextual elements like objects or characters (Sacripante et al., 2023). Consider the ‘vacuuming the seats’ subevent from the ‘car washing’ event. The gist requires depicting both a vacuum cleaner and its interaction with the car seats; omitting either changes the subevent’s fundamental meaning. If either element were absent (e.g., if only the vacuum was drawn without showing its interaction with the car seats, or if the vacuum was replaced with a brush), the drawing would receive a gist score of 0; similarly, if participants only depicted a generic car wash scene (like drawing a car with a person) or simply wrote down the cue word directly, the gist score was 0; if both elements were correctly depicted, it would receive a score of 1. Moreover, if participants’ drawings contained elements from other subevents, like a hose or soap, the gist was regarded as incorrect as it overlapped with another subevent. However, variations in how these elements were drawn (such as the type of vacuum, the style of the car seats, or whether the drawer included decorative details of the car interior) did not affect the gist score as long as the core elements and their relationship remained clear in depicting the act of vacuuming the seats. To assess coding agreement between coders, we directly compared each participant’s total detail counts between the two coders: the coders assigned identical total scores 79.6% of the time, and their total counts were within one detail of each other 97.6% of the time, demonstrating strong counting consistency with minimal scoring deviations. To assess statistical reliability, we used intraclass correlation coefficients (ICC) across the sum of all recalled details, which measures consistency in ranking participants relative to each other while accounting for differences between raters. The overall reliability was ICC(3,1) = 0.877, 95% CI = 0.870, 0.883, indicating good reliability (Koo & Li, 2016). At a finer level, reliability across detail categories and subevents was ICC(3,1) = 0.862, 95% CI = 0.859, 0.865, which also reflects good reliability.

To analyze duration judgments across days, we matched subevents based on their underlying meaning (gist). Coders examined participants’ drawings and paired each one with its corresponding subevent to verify accurate depiction. This careful matching process accommodated cases where participants recalled subevents in the wrong order. By confirming that matched pairs represented the same conceptual content across both testing days, we ensured that changes in duration judgments reflected genuine shifts in perceived duration rather than differences in the remembered subevents or errors in subevent identification. Interrater reliability for gist matching coding was high, with an overall weighted Cohen’s kappa of 0.96. Exact agreement between raters was 96.9% across all coded items.

After judging the gist accuracy of all participants’ drawings for a particular video event, coders checked the drawn items against a provided checklist. This checklist was compiled by having other coders analyze the videos and list all relevant details, including actions, characters, items, and environments. The coding process involved: (1) Verifying whether subevent items appeared correctly in participants’ drawings (1 = present, 0 = absent); (2) Evaluating any additional items not on the checklist as potential false memories, noting their actual presence or absence in the original event. We also applied consistent rules for accepting synonyms. The total accurate detail recalled is calculated by summing up all the details in the four categories: character, action, item and place. Inter-rater reliability for memory detail coding showed fair agreement between coders, with a correlation coefficient of *r* =.59. Exact agreement between coders occurred in 79.6% of cases, and agreement within ±1 detail was achieved in 97.6% of cases.

Six coders were involved, divided into three groups, each composed of two coders, to ensure inter-rater reliability. Each group was responsible for coding 10 videos or events. If the two coders disagreed, a third coder was consulted, and the final coded result was determined by majority decision. Inter-rater reliability is calculated by comparing the ratings of two coders within each group.

#### Behavioral data analysis

**Fig. 2:**
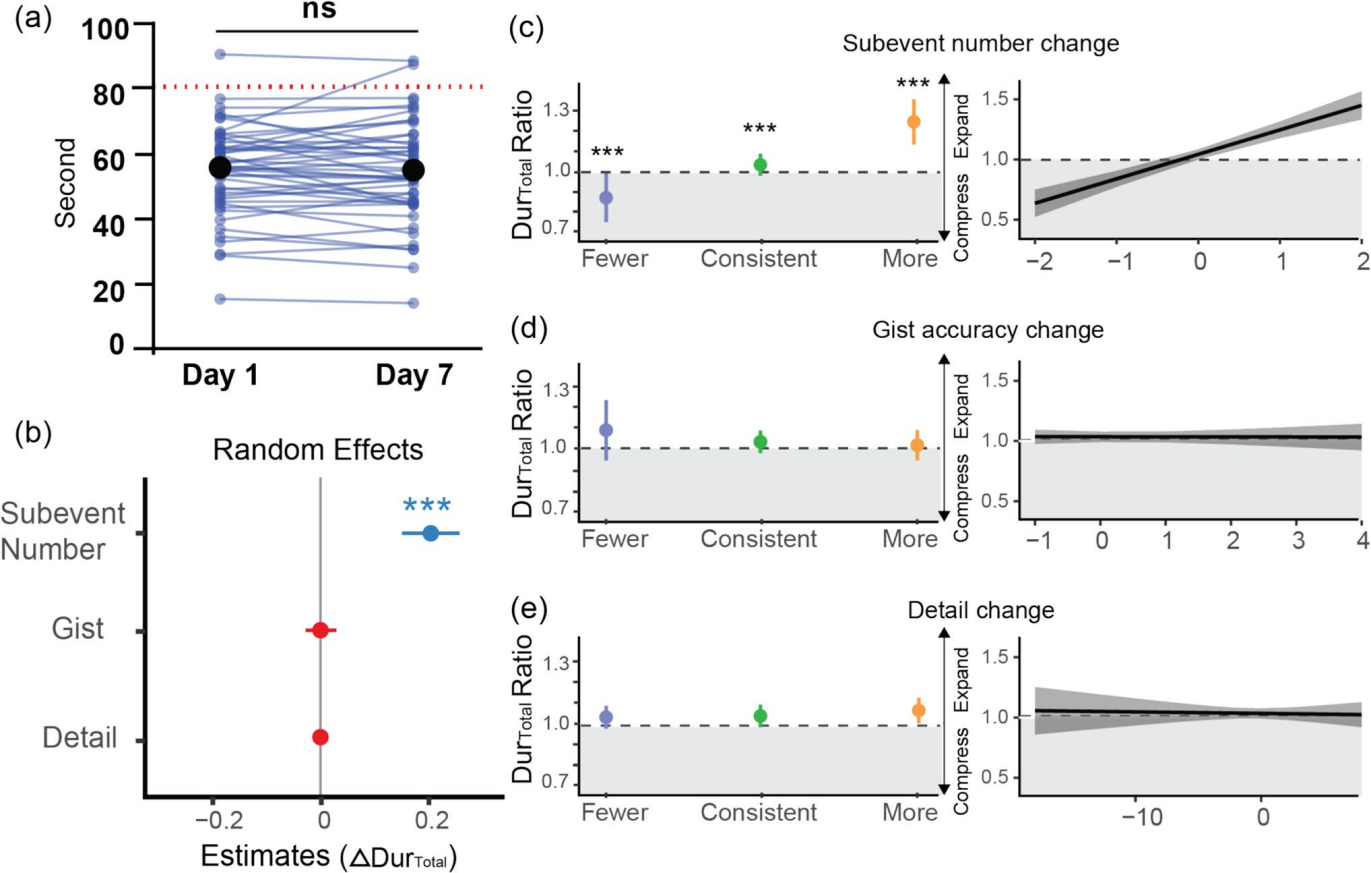
Event duration changes are only modulated by memory changes of recalled subevent number. **(a)** Changes in reported event duration among participants across days. Blue dots represent the mean reported duration for each participant. Black dots indicate the mean reported duration for all participants on each day. The red dotted line represents the actual event duration. **(b)** Main effect of across day change in recalled subevent number (*p* <.001), gist accuracy (*p* =.975) and recalled detail number (*p* =.826) on Dur_Total_ Ratio. **(c–e)** Plots of estimated Dur_Total_ Ratio across days by change of **(c)** recalled subevent number, **(d)** gist accuracy, and **(e)** details respectively. Left panel, data were grouped based on the changes of memory across days with Fewer (Blue), Consistent (Green) and More (Orange) from Day 1 to Day 7. Right panel, a continuous estimation of how unit changes on memory affect retrospective duration. The dashed line represents the grey area where Dur_Total_ Ratio = 1, indicating no compression. Grey area below 1 represents Dur_Total_ Ratio < 1, indicating compression on Day 7. Error bars and shaded areas indicate the 95% confidence intervals (CI). *** *p* <.001. (Color figure online)

**Fig. 3:**
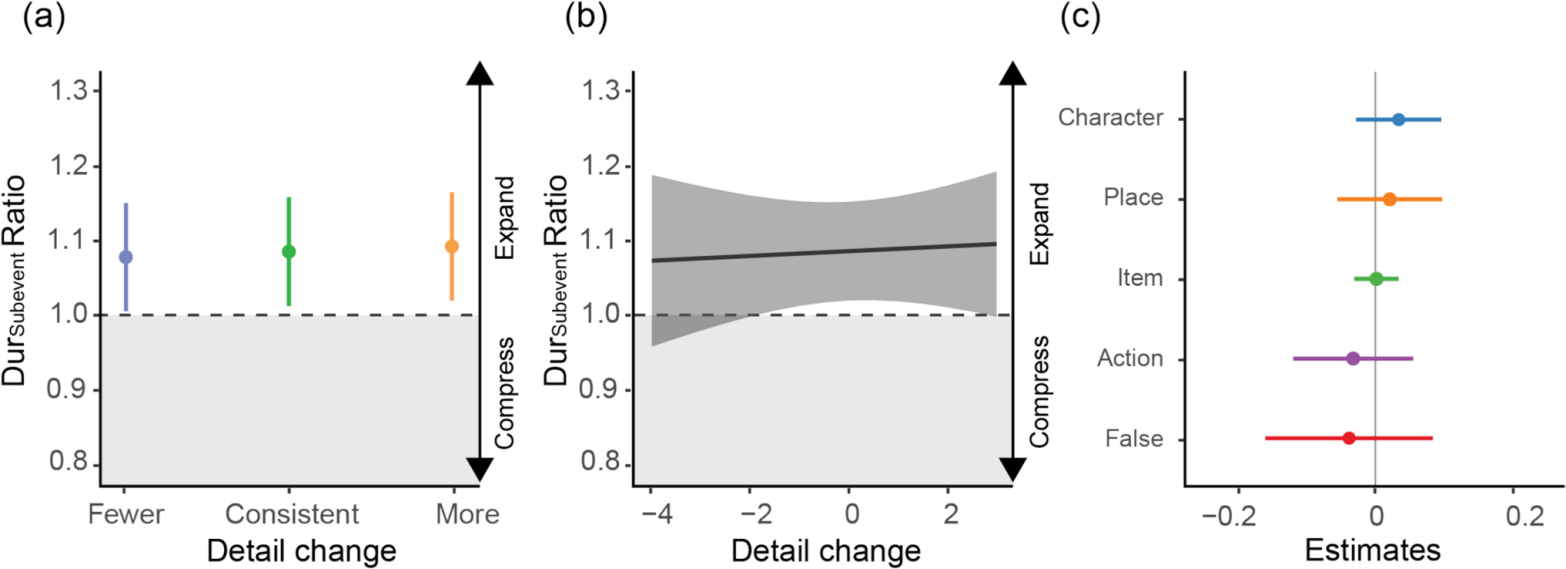
Subevent duration changes are not modulated by memory changes of recalled detail number. **(a)** Similar to Fig. 2e but for individual subevent. Fewer (Orange, *n* = 503 number of subevents), Consistent (Green, *n* = 497), More (Blue, *n* = 495). No significant effect is found (*p *values >.05). **(b)** Continuous estimation. **(c)** Predicting subevent Dur_Subevent_ Ratio by the change of detail per different categories (character, place, item, action, false memory item) across days. No significant main effect was found (*p* values >.05). For the frequency distribution of overall detail changes and by category, see Fig. S7. Error bars and shaded areas indicate the 95% confidence intervals. (Color figure online)

The statistical analyses and graphical representations were performed using R (Version 2023.03.3) and Prism (Version 9.4.1 for Windows). For the analysis of duration judgment changes across days, we employed linear mixed-effects models (LMMs) to account for participant-level random effects and video-specific variability at the trial level. Separate models were constructed to predict both changes in total duration judgment (Fig. 2) and subevent duration judgment across days as the dependent variables (Fig. 3).

We initialized our mixed models with a maximal random effects structure, including random intercepts and slopes for all within-subject and within-event experimental factors, to capture individual variation and minimize bias from unmodeled variance (see Table S3 and Table S2 for the experimental factors included and comparison of selected models). For model selection, we employed a backward selection approach, as recommended by Meteyard and Davies (2020), to mitigate Type 1 errors by preventing overfitting. Starting with the maximal model, we removed non-significant predictors and interactions stepwise using the R package buildmer (Voeten, 2023), which relies on the Akaike information criterion (AIC) for elimination decisions. To address the risk of Type 2 errors from oversimplification, we then complemented this with a forward selection approach, adding theoretically relevant predictors step by step to a minimal model manually. To assess model fit and select the optimal structure, we used Bayes factors (BF01) for model comparison (results reported in Table S2). BF01 quantifies the relative evidence for competing models, favoring simpler structures unless strong evidence justifies complexity, thus reducing Type 1 error risk (van Doorn et al., 2023). This dual strategy ensures a robust balance between complexity and parsimony.

To investigate the factors influencing duration judgments on single Day 1 and Day 7, separate LMMs were constructed for individual day’s data. The LMM was defined as:

$$\text{Day }1/\text{Day }7\text{ reported duration}\sim \text{reported number of subevents}+\text{gist accuracy}+\text{the number of correctly recalled details}+\text{recall order accuracy}+\text{encoding order}+\text{test order}+\left(1|\text{Subject ID}\right)+\left(1|\text{Event ID}\right)$$

Specifically, the encoding order refers to the sequence in which videos were presented during the encoding session, test order refers to the sequence of testing during the retrieval session, and recalled subevent order accuracy refers to whether participants correctly recalled subevents in the same order as originally presented in the video, coded by sorting participants’ recalled gist and matching it the original video sequence. The encoding order, test order and recall order accuracy were later excluded in the single-day LMM (Fig. S3) due to insignificant effects on predicting single-day duration judgments.

Prior to model fitting, we assessed collinearity among our key predictors (subevent number, gist accuracy, and number of details recalled) using Variance Inflation Factor (VIF) analysis and correlation coefficients (O’Brien, 2007). VIF values for all predictors were well below the threshold of 5 (ranging from 1.069 to 1.238), and correlations between predictors were modest (|*r*| < 0.31), indicating minimal collinearity concerns across all models (see Tables S4 and S5).

All linear mixed effects models (LMMs) were conducted using the lme4 package (Bates et al., 2015). The significance levels for fixed and random effects, as well as all Likelihood ratio tests used to compare model fits, were assessed using the ‘anova’ function in the *lmerTest* package (Kuznetsova et al., 2017).

## Results

Despite spending considerable time on watching multiple videos, participants remained sensitive to actual subevent duration differences on both Day 1 and Day 7. Specifically, participants successfully distinguished between 20-second and 40-second subevents (Day 1, *t*(47) = −5.253, *p* <.001, *d* = 0.758; Day 7, *t*(47) = −5.31, *p* <.001, *d* = 0.766; Fig. S1). Consistent with prior research, our findings also reveal significant temporal compression compared with the actual duration of the entire event, starting on Day 1, *t*(47) = −10.956, *p* <.001, *d* = 1.581 (Hicks et al., 1976; MacDonald, 2014; Tsao et al., 2022; Yarmey, 2000; Zakay & Block, 2004; Fig. 1e).

### Duration judgments vary by memory level during immediate or delayed tests

We investigated whether immediate (Day 1) and delayed (Day 7) judgments of whole event duration depend on event structure and content recall. We employed a linear mixed model (LMM) to identify the main influencing factors while accounting for variability among participants and events as suggested in previous studies (Lositsky et al., 2016; Safi et al., 2024). Encoding order did not affect reported duration on Day 1, *F*(1, 666.89) = 0.007, *p* =.934, η^2^ < 0.001, and Day 7, *F*(1, 668.53) = 0.140, *p* =.708, η^2^ < 0.001. Test order similarly showed no significant effect on Day 1, *F*(1, 666.50) = 0.959, *p* =.328, η^2^ = 0.001, and Day 7, *F*(1, 666.52) = 2.892, *p* =.089, η^2^ = 0.004. Accuracy of recalled subevent order also had no effect on Day 1, *F*(1, 659.57) = 0.811, *p* =.368, η^2^ = 0.001, and Day 7, *F*(1, 616.41) = 0.844, *p* =.359, η^2^ = 0.001. Therefore, we excluded order as a fixed factor in our final models (see Methods).

The analysis revealed that the number of recalled subevents significantly influenced total duration reported. Specifically, for each additional subevent recalled, participants’ reported durations were, on average, 12.23 s longer on Day 1, *F*(118.251, 1) = 243.531, *p* <.001, η^2^ = 0.67, and 11.06 s longer on Day 7, *F*(168.751, 1) = 330.939, *p* <.001, η^2^ = 0.66. Additionally, neither gist accuracy—Day 1, *F*(213.144, 1) = 0.230, *p* =.632, η^2^ = 0.001; Day 7, *F*(190.831, 1) = 3.155, *p* =.077, η^2^ = 0.02—nor the total recalled number of details—Day 1, *F*(59.279, 1) = 2.232, *p* =.141, η^2^ = 0.04; Day 7, *F*(55.903, 1) = 0.013, *p* =.910, η^2^ < 0.001—contributed significantly to the reported total duration (Fig. S3). Thus, event duration judgment appears to involve recalling directly the number of subevents with minimal regard to the retrieval of event content.

Notably, the duration judgment of an entire event is not merely the sum of all reported subevent durations, despite their significant correlation (Day 1: *r* =.795, *p* <.001; Day 7: *r* =.602, *p* <.001; see Fig. S4). Specifically, the sum of subevent duration was significantly longer—Day 1: *t*(47) = −3.134, *p* =.003, *d* = −0.453; Day 7: *t*(47) = −3.01, *p* =.004, *d* = −0.434—suggesting different strategies for judging event versus subevent durations.

To investigate whether subevent duration judgments depend on content memory recall, we ran an LMM with subevent gist accuracy and number of recalled details as fixed factors, and participant and event as random factors. Results suggest that subevent duration judgments depended on the gist accuracy—Day 1, *F*(2,1765.06) = 3.762, *p* =.023, η^2^ = 0.004; Day 7, *F*(2,1712.41) = 3.468, *p* =.031, η^2^ = 0.004—and the number of recalled details—Day 1, *F*(1,1571.27) = 22.520, *p* <.001, η^2^ = 0.01; Day 7, *F*(1,1618.20) = 8.313, *p* =.004, η^2^ = 0.005 (see Fig. S5). These results suggest judgments for duration judgments might involve distinct processes depending on the event structure: whole event durations are influenced by event structure memory, while subevent durations rely on the richness of episodic content recall.

### Across days: Unique role of event boundary on event duration judgments

After encoding, memory undergoes transformation over time. These natural memory changes provide a unique opportunity for us to probe how durational information may be related to stored memories. We first confirmed memory changes occurred by comparing Day 1 and Day 7 memory performance using paired t-tests. We observed an overall decline in memory performance across all memory levels. Specifically, the accuracy of recalled number of subevents dropped significantly, *t*(47) = 4.051, *p* <.001, *d* = 0.512, with the occurrences of both forgetting (79 out of 593 occurrences) and false memories, where participants recalled more subevents than actually occurred (52 out of 593 occurrences), observed on Day 7. For event content, we also observed significant forgetting tendency across days, including gist, *t*(47) = 4.00, *p* <.001, *d* = 0.411, and the number of accurately recalled details, *t*(47) = 5.842, *p* <.001, *d* = 0.843 (Fig. 1e).

We next assessed the change in reported total event duration across days, quantified as the ratio of Day 7 to Day 1 (Dur_Total_ Ratio), with a value of 1 indicating consistent duration judgment, > 1 and < 1 values indicating expanded and compressed duration judgments relative to Day 1, respectively. We employed LMM to test whether this ratio can be explained by changes in recalled subevent number, gist accuracy and number of recalled details across days as fixed factors, and participants and event as random factors. Consistent with the above results for single days, Dur_Total_ Ratio was significantly affected by changes in recalled subevent number, with temporal compression associated with forgetting whereas temporal expansion linked to falsely inserted subevents, *F*(1, 692.32) = 55.34, *p* <.001, η^2^ = 0.07 (Fig. 2). However, Dur_Total_ Ratio remained unaffected by changes in overall gist accuracy, *F*(1, 491.28) = 0.001, *p* =.975, η^2^ < 0.001, and the number of recalled details, *F*(1, 669.02) = 0.049, *p* =.826, η^2^ < 0.001. In fact, the fit of this model did not significantly differ from a simpler LMM with only a single fixed factor—change in the number of subevents (*AIC* = 353.74; *BIC* = 376.67; *LR* = 0.04; *p* =.98 over the three-factor model including gist accuracy and recalled detail; see Table S2). The observation that Dur_Total_ Ratio parallelled the change in the number of subevents recalled provides additional and direct support to our conclusion from single-day analysis that event duration judgment depends on memory for event structure and not event content.

### Across days: Subevent duration judgment change is dissociated from memory change

Next, we focused on individual subevents and explored whether changes in duration judgments are influenced by alterations in subevent-specific memories. Since participants may have forgotten multiple subevents on Day 7, we identified subevents that were correctly recalled on both Day 1 and Day 7 based on their gist (see Methods for matching details). Then, we calculated the Dur_Subevent_ Ratio for each pair (Day 7/Day 1). For these matched subevents, we observed a significant change on recalled details across days, including forgotten details and false memories—one-sample *t* test; *t*(47) = 25.35, *p* <.001, *d* = 3.66. However, despite these substantial memory changes, we found no significant differences in overall Dur_Subevent_ across days (*p* =.999; Fig. S6). To directly test whether changes in recalled details predict Dur_Subevent_ judgments, we employed a linear mixed model (LMM) accounting for random effects of participants and events. The results confirmed our initial finding: despite the general reduction in recalled details, participants’ Dur_Subevent_ judgments remained stable (*β =* −0.01, *SE* = 0.03), *t*(1458.74) = −0.38, *p* =.702 (Figs. 3a, b). Likewise, recalling more correct details on Day 7 than on Day 1 did not significantly alter duration judgments (*β* = 0.01, *SE* = 0.03), *t*(1475.13) = 0.24, *p* =.811. Furthermore, when comparing this model to the baseline model, which excluded memory level (i.e., detail recall) as a fixed factor, the results showed no significant improvement (*LR* = 0.1, *p* =.752; see Table S3 for model comparison). This suggests that the changes in recalling event details did not significantly influence change of judgments of subevent duration over time.

While changes in subevent duration judgments appear independent of the total details recalled, certain types of details might still have an impact. Action-related details, for instance, may be more significant predictors of subevent duration than static items, as perceived speed influences time perception (Burt, 1999; Burt & Popple, 1996; Mioni et al., 2015; Sasaki et al., 2013). To explore this, we further classified participants’ recalled details in our previous LMM into distinct categories, including character, action, item, place, and falsely recalled details. Although character and place details predicted duration on individual days (Fig. S8), none of these categories significantly predicted changes in Dur_Subevent_ Ratio across days: change in character, *F*(1,1474)=1.122, *p* =.290, η^2^ = 0.001; action, *F*(1,1449.8) = 0.274, *p* =.601, η^2^ < 0.001; item, *F*(1,1461.9)= 0.001, *p* =.978, η^2^ < 0.001; place, *F*(1,1462.9) = 0.292, *p* =.589, η^2^ < 0.001, and; falsely recalled item, *F*(1,1452.8) = 0.118, *p* =.731, η^2^ < 0.001 (see Fig. 3c and Table S3).

### Subevent duration judgments averaged across days

Could decreased recall details over days suggest memory schema formation, leading to generalized duration judgments, thus explaining the minimal impact of subevent content changes on subevent judgments above? To test this, we investigated whether participants tended to perceive each subevent duration more uniformly on Day 7 than on Day 1. We calculated the deviation of each participant’s reported subevent duration from their average reported subevent duration for each day, and analyzed how these deviations changed across all subevents between Day 1 and Day 7. Interestingly, participants’ reported subevent durations tended to converge towards their average over days, *t*(47) = 5.32, *p* <.001, *d* = 0.768 (Fig. S9). Moreover, there is a significant decrease in the difference in duration judgments between 20 s and 40 s subevents across days, paired *t* test, *t*(47) = − 2.02, *p* =.04, *d* = 0.291. All these suggest that the subevent duration tends to become more similar as time passes.

## Discussion

Human experience is continuous, yet it can be structurally segmented into meaningful events and further into subevents during encoding and retrieval. We investigated how changes in event structure and content memory, including event boundaries, subevent gist, and details, influence retrospective duration judgment. We employed a within-subject test-retest design, enabling simultaneous measurement of both memory changes and duration judgments. To ensure well-defined event structure, we used video clips with sharp boundaries, validated by participants’ key presses during video watching. Our findings indicate that first, people’s judgments of event duration rely primarily on event structure memories, that is their recall of the subevent number. Second, the judgment of subevent duration depends on event content memory, including the recalled gist accuracy and detail number, with a tendency towards averaging across days.

Our results suggest that judgments of total event duration may rely more heavily on recalling structural boundaries, but not specific event details, aligning with spatial navigation research showing that more cognitive boundaries (i.e., turns) reduce temporal compression during route recall (Bonasia et al., 2016; Brunec et al., 2020). One possible explanation is that event boundaries serve as retrieval cues that facilitate access to temporal information stored in memory (Michelmann et al., 2023). When recalling an event, people may use these structural markers to retrieve temporal information, rather than reconstructing duration from specific content details. Supporting this possibility, our results suggested a mild negative correlation between the number of subevent and details recalled (Day 1: *r* = −0.16; Day 7: *r* = −0.19). One potential mechanism underlying these findings is that duration information may be stored not as exact numerical values, but as relational representations that preserve comparative information about event lengths and temporal relationships (Baldassano et al., 2018; Khemlani et al., 2015). During judgment, these relational representations can be converted into specific duration estimates, while finer details such as subevent gist accuracy and recalled details may be neglected in this process. Future studies could further explore how this conversion process is accomplished.

Regarding duration judgments of subevent, we found that the underlying processes may differ from those used for entire events. Specifically, subevent duration judgments rely on memories of the subevent content, despite their relatively small effect. This distinction is further highlighted by our across-day analyses, which showed that changes in memories for event structure influenced event duration judgments, whereas alterations in memories related to event content did not significantly impact subevent duration judgments. This finding suggests that the event segmentation model (Block, 1992; Bonasia et al., 2016; Brunec et al., 2020; Ornstein, 1969; Zakay & Block, 2004), which posits that people perceive duration retrospectively by recalling discrete segments marked by significant changes in context, activity, or goals, may not universally apply to duration judgments of subevents that contain no further segments. Nevertheless, while event duration judgments remained consistent across Day 1 and Day 7, subevent duration judgments tended to average over time. This averaging tendency may be due to increased similarity among subevents across different events as experiences within similar contexts often result in analogous representations, thus diminishing the distinctiveness of each video over time and facilitating generalization of durational schemas (Baldassano et al., 2018; Reagh & Ranganath, 2023). Future studies can explore how memory schematization may affect duration judgment.

These findings prompt reflection on whether duration judgment is a reconstructive process based on memory or a direct recall of temporal information. Some researchers suggest that duration judgment is influenced by the volume of retrievable memory content, particularly by boundaries marked by contextual changes, as described by the storage size and event segmentation models (Block, 1992; Ornstein, 1969; Zakay & Block, 2004). In contrast, other studies argue that duration is directly encoded and functions independently of memory accuracy. For instance, individuals accurately judged durations of past public events even with imprecise memories (Burt, 1992; Burt & Kemp, 1991; Friedman, 1993). Our study suggests that the approach to judging duration is mixed and contingent on the event’s structure and complexity. For complex events, duration judgments rely on event structure, providing efficient duration judgment consistent with the minimal effort hypothesis, requiring no additional effort to access specific event content and details (Stojić & Nadasdy, 2024). In contrast, for subevents with no internal boundaries, duration judgment might be based on the average impressions of all subevent duration, where minor adjustments are made through episodic details recall. Future studies could explore how manipulating event boundaries after encoding influences duration judgments, especially given the flexibility in event boundary retrieval (Hohman et al., 2013).

In conclusion, people’s duration judgments vary by event complexity, with whole events relying on structural memories while subevents appear to be influenced by content memories. Preserving the accuracy of event durations is crucial, as it shapes our perception of the past and supports precise time estimations for future planning. Indeed, individuals with memory impairments, such as those caused by brain injury or neurodegenerative conditions like Alzheimer’s disease, often experience distorted time perception (El Haj & Kapogiannis, 2016; El Haj et al., 2013; Mioni et al., 2014). These temporal inaccuracies not only disrupt recollections of the past but may also affect one’s self-identity (Piras et al., 2014). Our data suggests a novel perspective, indicating that time perception may be more closely linked to event structure rather than solely depending on memory accuracy or richness. Therefore, enhancing the memory of event structure after encoding could actively preserve duration perception. Future research should explore whether strategies focusing on event structure might help maintain subjective retrospective duration among healthy and clinical population.

## Supplementary Information

Below is the link to the electronic supplementary material.

Supplementary file1 (DOCX 808 KB)

## Data Availability

Data is available on OSF: https://osf.io/m43yh/?view_only=8f93a14ba974440f91c3be8a4e9c99ad

## Code availability

Code is available on OSF: https://osf.io/m43yh/?view_only=8f93a14ba974440f91c3be8a4e9c99ad
