# Supplementary material for: Retrospective duration judgments of naturalistic events depend on memories of event boundaries
Source: Psychon Bull Rev. 2026 Jan 5;33(1):33. doi: 10.3758/s13423-025-02833-z (PMC12769706; doi:10.3758/s13423-025-02833-z)
Supplement: Supplementary file 1 — Supplementary file1 (DOCX 808 KB) [file 13423_2025_2833_MOESM1_ESM.docx]

**Supplementary**


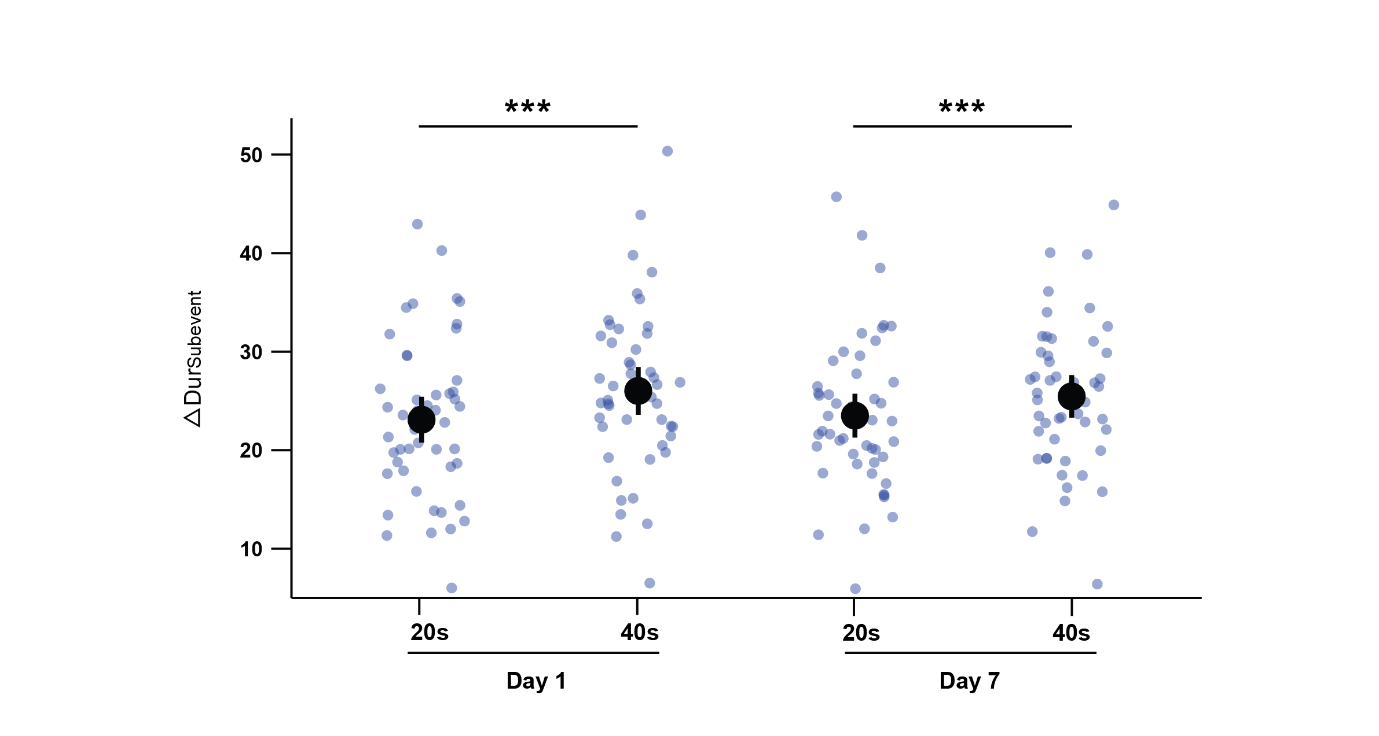

Fig. S1. Reported duration for 20-s and 40-s subevent on both Day 1 and Day 7. Significant differences were found on between 20-s and 40-s subevents on Day 1 (*t*(47) = -5.253, *p* < .001, *d* = 0.758) and on Day 7 (*t*(47) = -5.31, *p* < .001, *d* = 0.766). Blue dots represent the mean reported duration for each participant. Black dots indicate the mean reported duration for all participants on each day. Error bars indicate the 95% confidence intervals. Statistical significance is denoted by stars, with *** indicating *p* < 0.001.


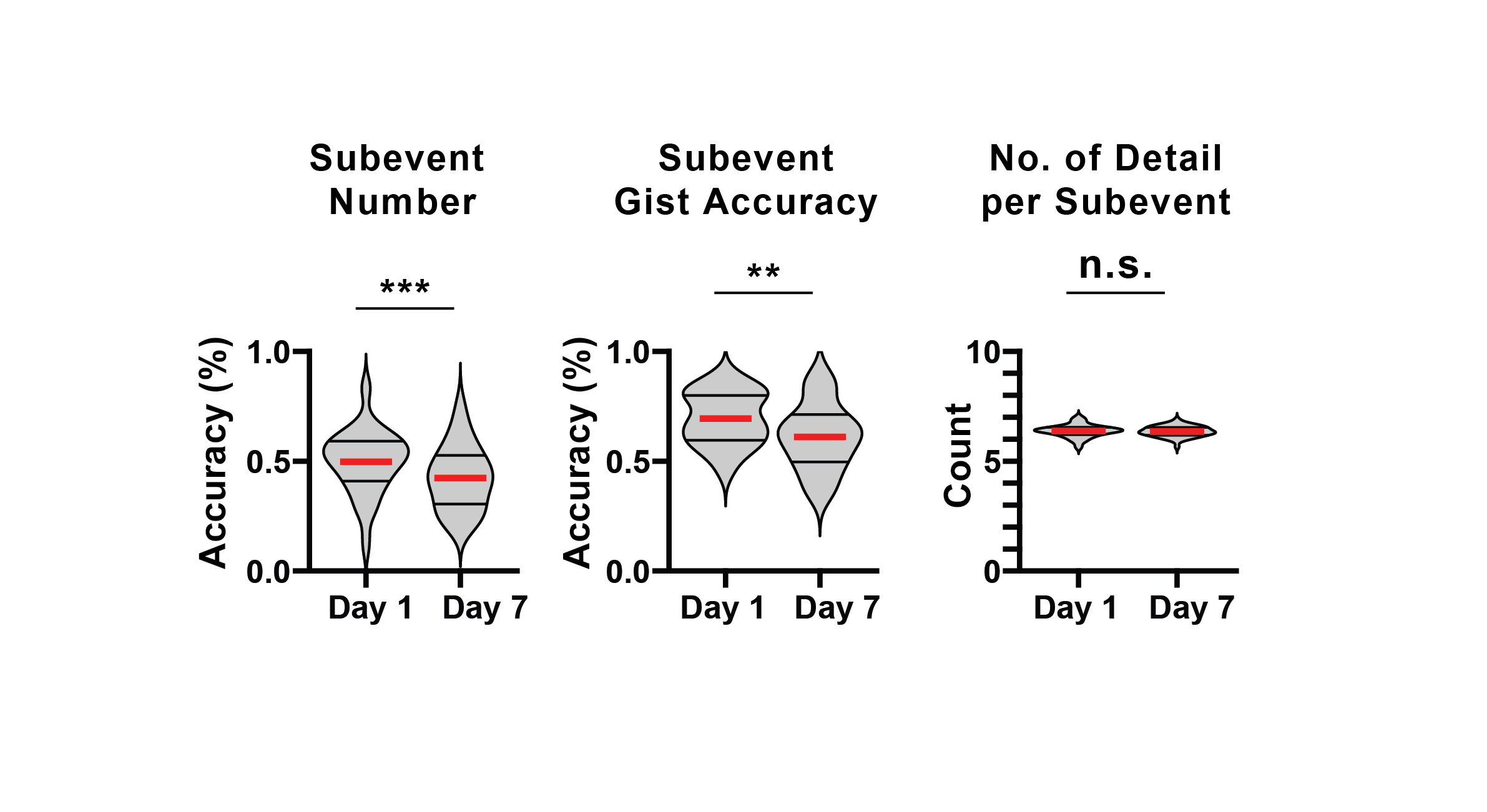


Fig. S2. Memory performance across days (before filtering). Left, for the reported subevent number. Middle, for the recalled gist accuracy. Right, for the number of details. Significant differences were found for changes in the number of subevents (*t*(47) = 5.232, *p* < .001, *d* = 0.755) and subevent gist accuracy (*t*(47) = 6.051, *p* < .001, *d* = 0.601), indicating forgetting. However, no significant difference was found for the number of details per subevent across days (*t*(47) = 0.475, *p* = .637, *d* = 0.073). Statistical significance is denoted by stars, with ** indicating *p* < 0.01, and *** indicating *p* < 0.001.


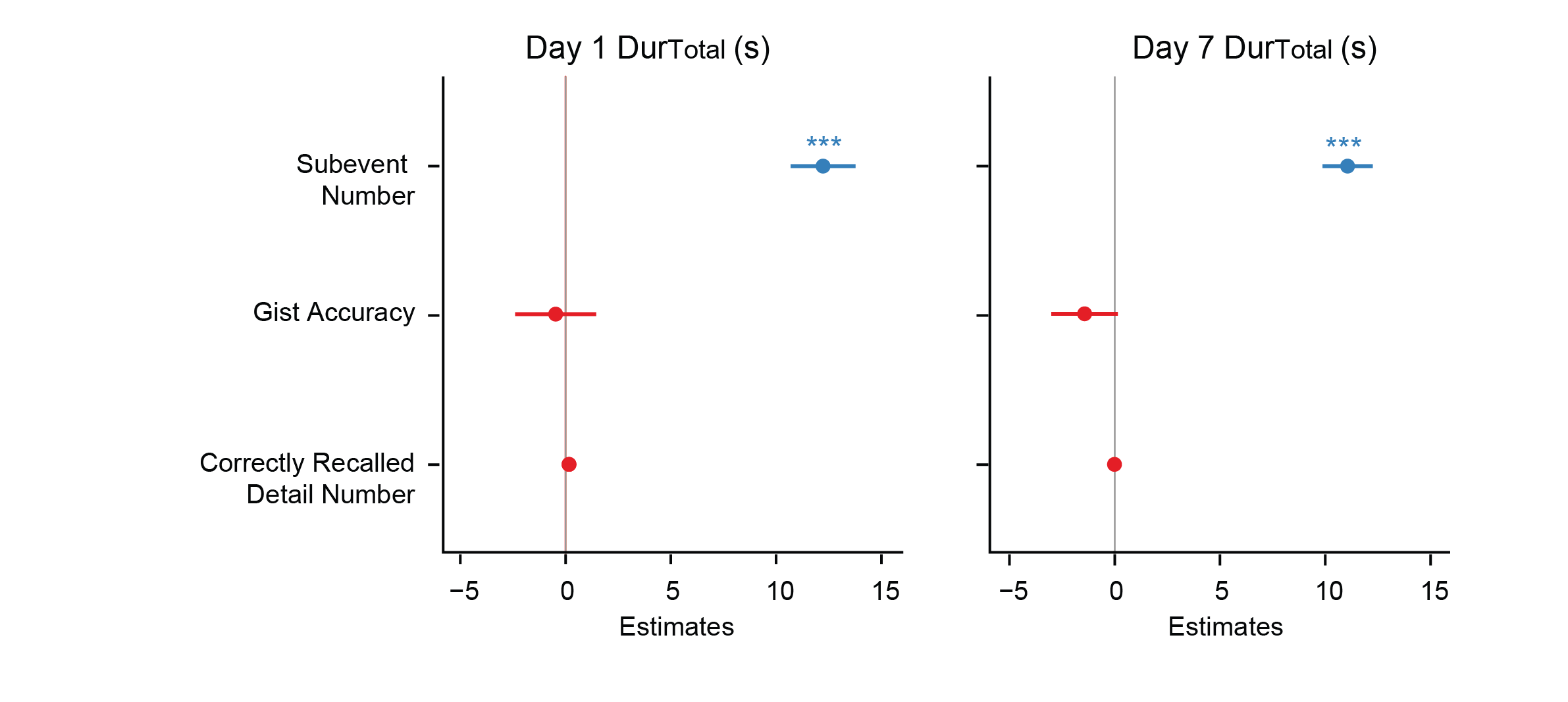


Fig. S3. LMM results for Day 1 and Day 7 reported Dur_Total_ including subevent number, gist accuracy, and the number of correctly recalled details, with random effects for participant and event. Only the number of recalled subevents significantly predicts the Dur_Total_ on both Day 1 (*F*(1,118.251) = 234.531, *p* < .001, *η²* = 0.67) and Day 7 (*F*(1,168.751) = 330.939, *p* < .001, *η²* = 0.66). Statistical significance is denoted by stars, with *** indicating *p* < 0.001.


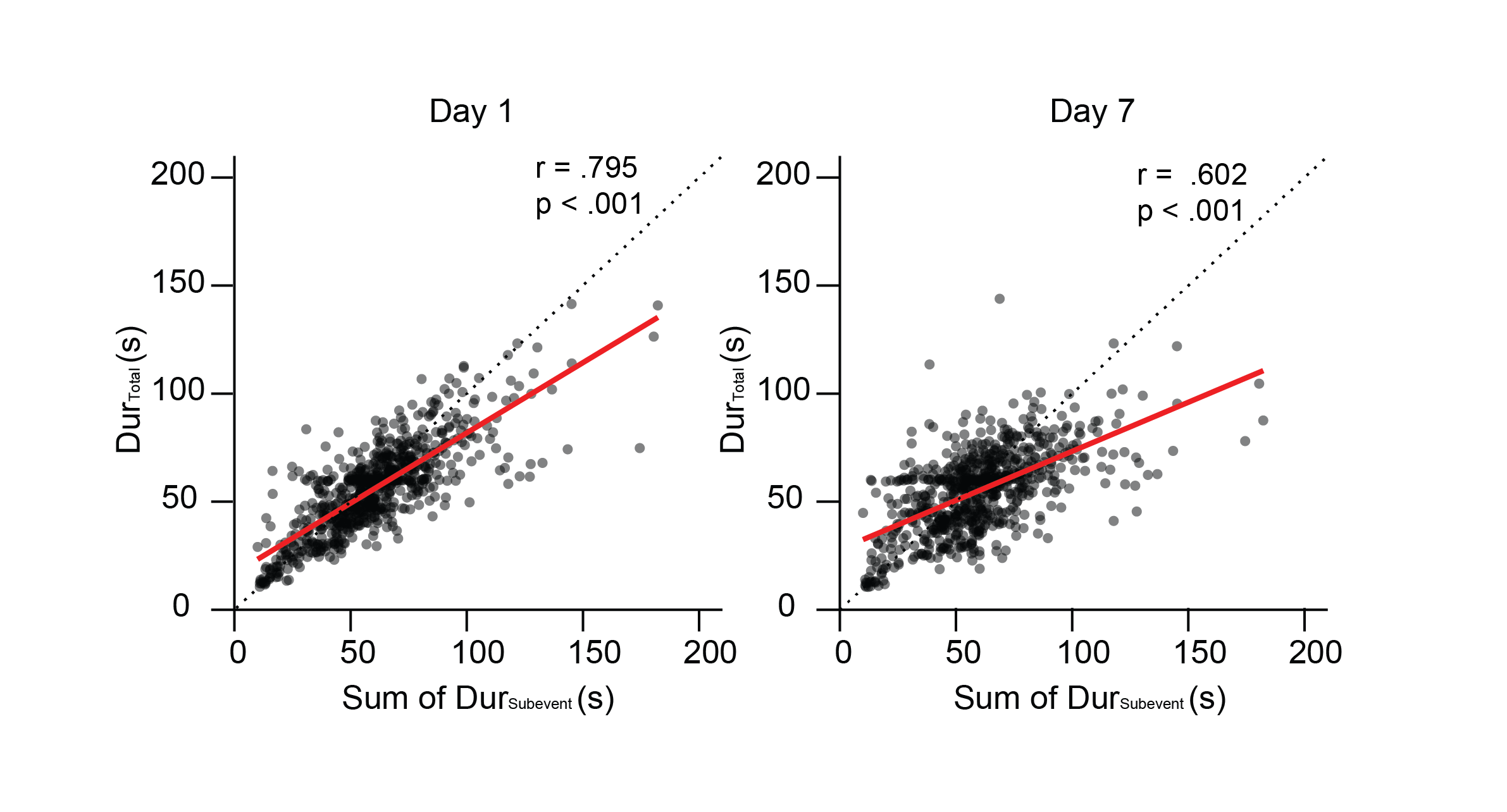


Fig. S4. Significant positive correlations between the sum of all reported Dur_Subevent_ and Dur_Total_ on both Day 1 (*r* = .795, *p* < .001) and Day 7 (*r* = .602, *p* < .001). The red line represents the best-fit regression line, while the black dotted line indicates the diagonal. Each dot represents one video trial.


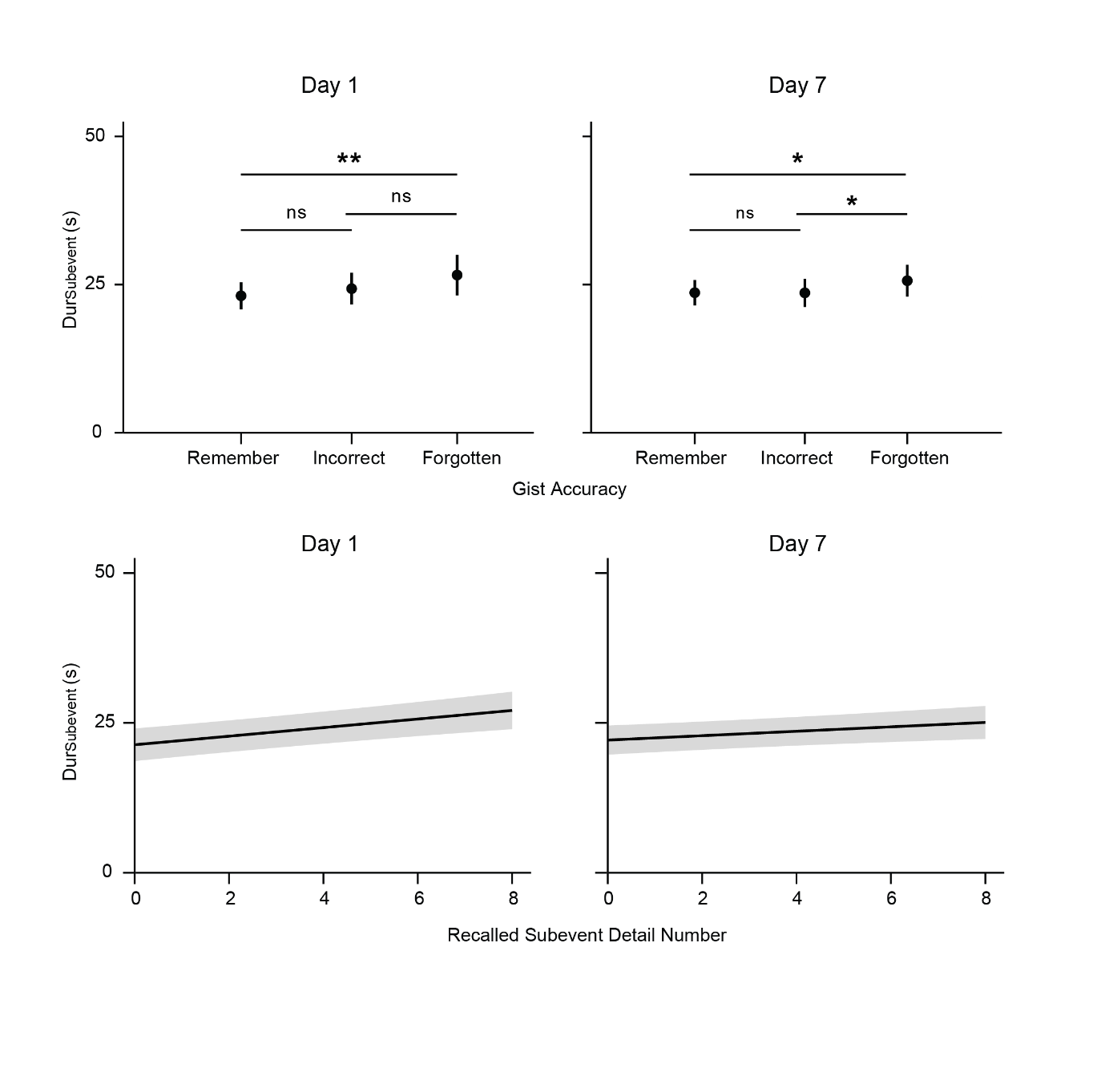


Fig. S5. Predicted Dur_Subevent_ in relation to subevent gist accuracy and subevent detail number on Day 1 and Day 7. Linear mixed-effects model (LMM) results for Dur_Subevent_ include subevent gist accuracy, original duration, correctly recalled detail number, and day as fixed factors, with random effects for participants and events. Both gist accuracy and detail number were significant predictors of Dur_Subevent_ on Day 1 (*F*(2,1765.06) = 3.762, *p* = .023, *η²* = 0.004 and *F*(1,1571.27) = 22.520, *p < .001*, *η²* = 0.01 respectively) and on Day 7 (*F*(2,1712.41) = 3.468, *p* = .031, *η²* = 0.004 and *F*(1,1618.20) = 8.313, *p* = .004, *η²* = 0.005 respectively). Error bars and shaded areas indicate the 95% confidence intervals. Statistical significance is denoted by stars, with * indicating *p* < 0.05 and ** indicating *p* < 0.01.


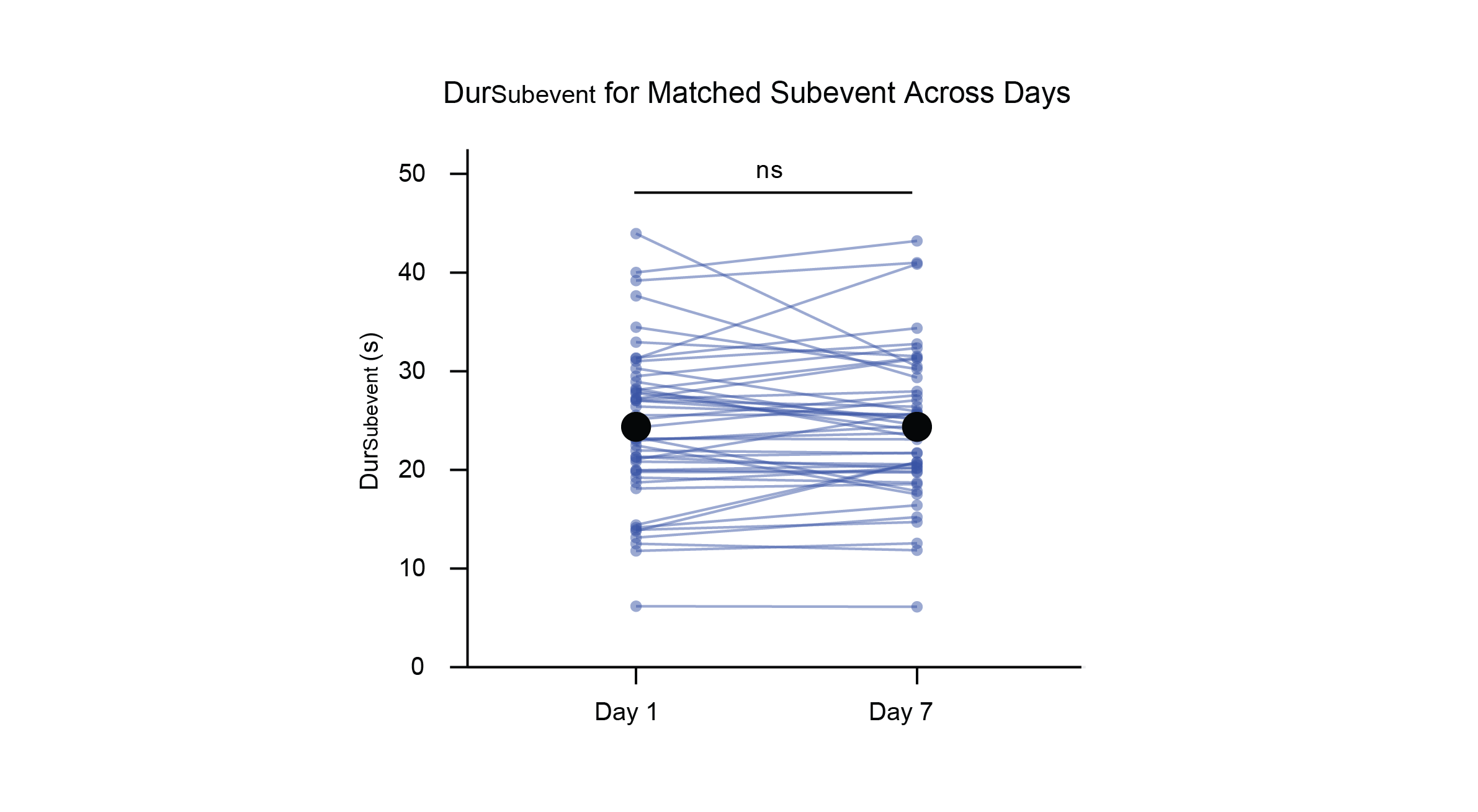


Fig. S6. Dur_Subevent_ on Day 1 and Day 7 by participants for matched subevents in Fig. 3. A paired t-test indicated insignificant differences between the two days (*t*(47) = -0.002, *p* = .999, *d* < 0.001). Blue dots represent the mean Dur_Subevent_ for each participant, while black dots indicate the overall mean Dur_Subevent_ across all participants for each day.


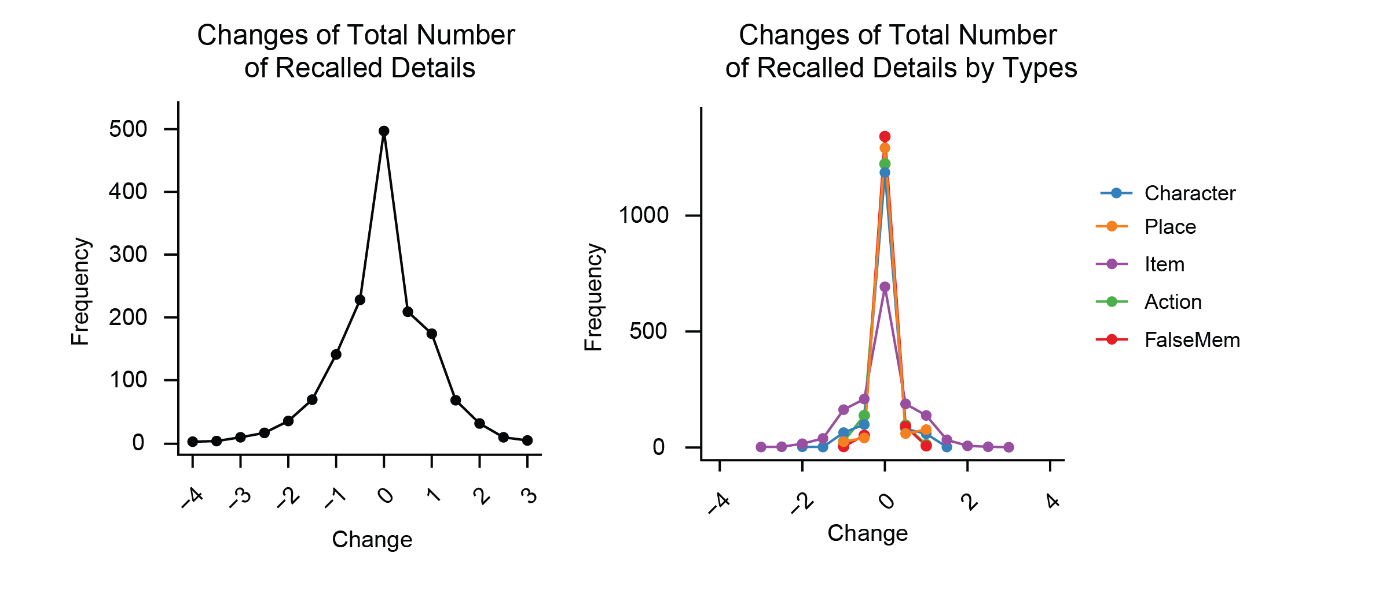


Fig. S7. Left panel, the distribution of detail number changes in Fig. 3. Right panel, the distribution of the number of detail changes, color matched with the detail types in Fig. 3­­­c) (*n* = 1495).


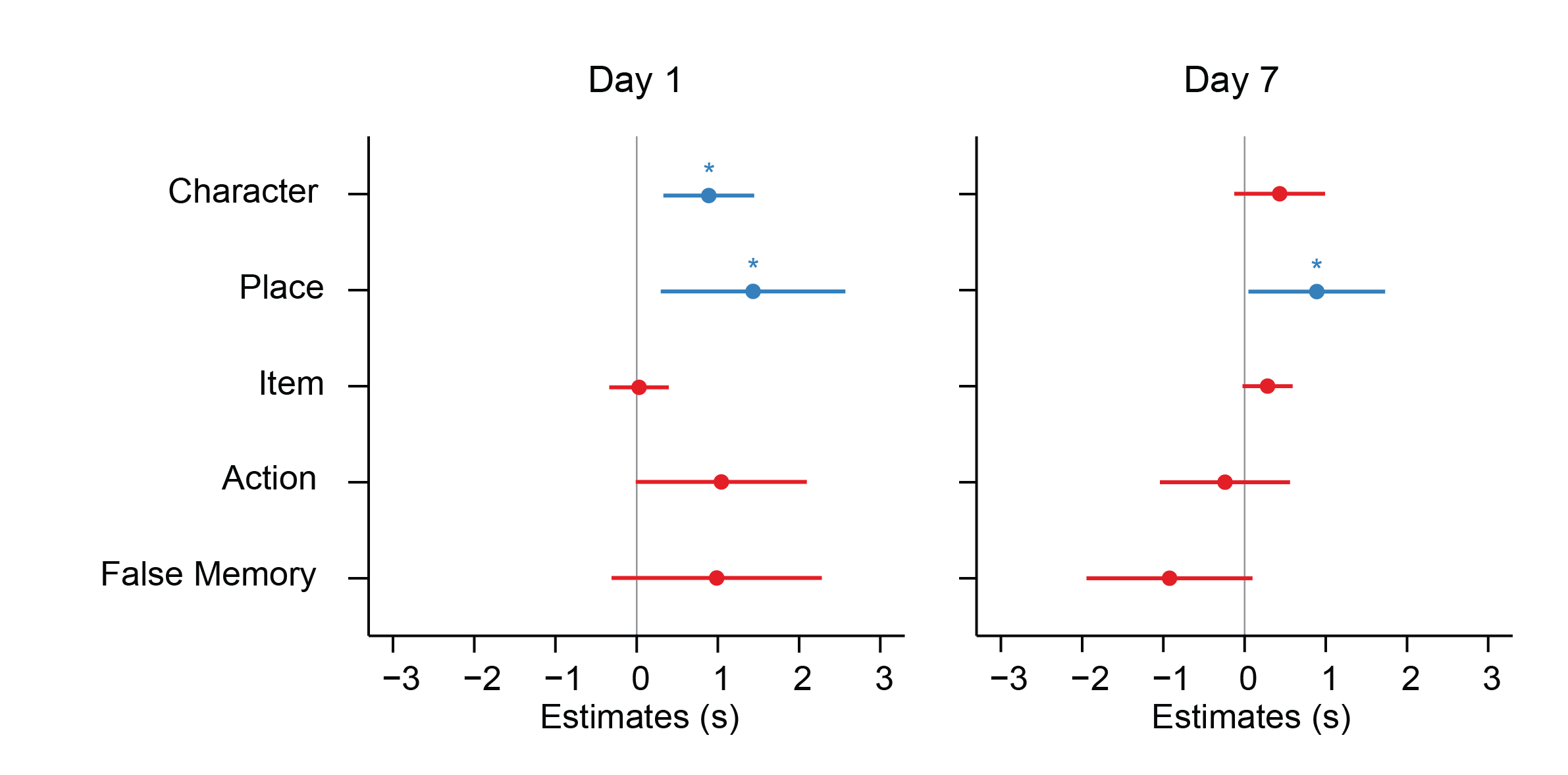


Fig. S8. The main effect of different types of detail on Dur_Subevent_. LMM performed on all Dur_Subevent_ with including subevent gist accuracy, original duration, different types of detail recalled and day as fixed factors, and random effects for participant and event. Subevent durations were significantly predicted by details related to characters (*F*(1, 1440.4) = 6.364, *p* = .012, *η²* = 0.004) and places (*F*(1, 1673.4) = 6.121, *p* = .013, *η²* = 0.004) on Day 1, and by places (*F*(1, 1667.2) = 4.303, *p* = .038, *η²* = 0.003) on Day 7. Statistical significance is denoted by stars, with * indicating *p* < 0.05.

_
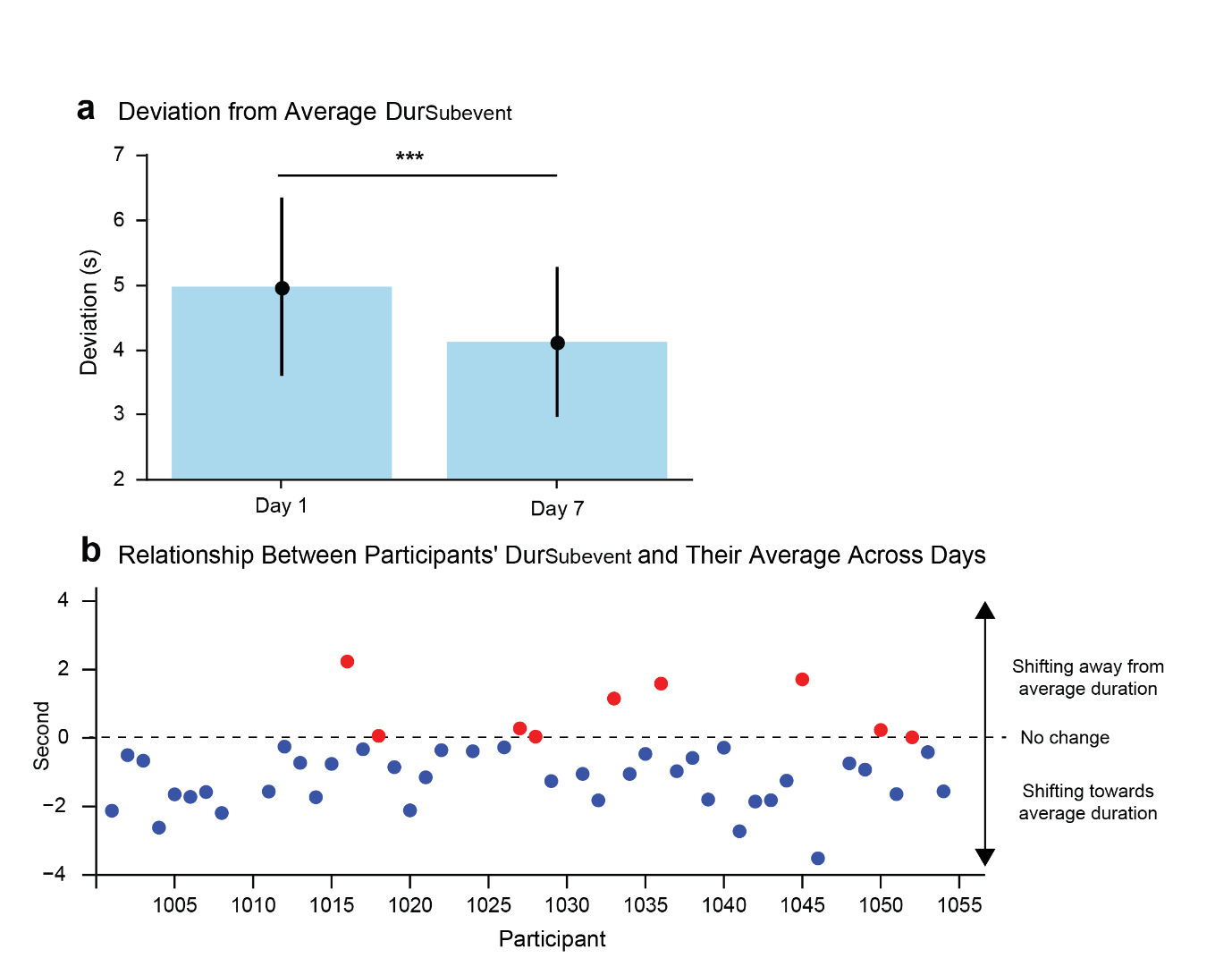
_

Fig. S9. Plots of participants’ reported subevent duration deviated from the average. The average duration was calculated for all subevents across all events for each participant. (a) Participants’ reported subevent durations showed a significant decrease in deviation, converging towards their average over the days (*t*(47) = 5.32, *p* < .001, *d* = 0.768). (b) Individual participants’ changes in mean deviation. Dots colored blue are below the dotted line of zero, indicating a tendency shifting toward the mean on Day 7. Dots colored red represent participants with a tendency shifting away from the mean. Error bars and shaded areas indicate the 95% confidence intervals. Statistical significance is denoted by stars, with *** indicating *p* < 0.001.

|  | Day 1 | Day 7 |
| --- | --- | --- |
| Participants exclusion due to poor performance (participant no.1010) | 30 | |
| Trials with incorrect subevent indication during initial encoding stage (including incorrect number of pressings, pressing before event boundary) | 243 |  |
| Trials where participants reported having seen the video before | 15 |  |
| Trials where participants indicated they had completely forgotten everything about the trial | 54 | 42 |
| Trials with no response during the total duration report | 22 | 25 |
| Trials indicated skip during the total duration report | 17 | 18 |
| Trials in which participants responded with ‘I forgot’ while reporting the number of subevents | 5 | 5 |
| Trials which participants did not provide an answer within the time limit during the subevent number report | 37 | 28 |
| Trials exhibiting unusual temporal compression of less than 10 seconds for total duration judgment | 4 | 2 |
| Trials where any single subevent duration exceeded the total duration, indicating possible data entry errors | 76 | 52 |
| Two videos, video number 9 and 18, were replaced due to ambiguous event boundary identification observed in the initial experimental phase | 16 | |
| Total trial left (remaining trial/ total number of trials) | 817/1440 | |

Table S1. Exclusion Criteria and Trial Attrition. The number is the excluded trials based on pre-defined criteria for Day 1 and Day 7 of the experiment. The categories listed represent the reasons for trial exclusion. Note that some trials may have been excluded for multiple reasons (i.e., overlapping exclusions), therefore the sum of exclusions in each column will not necessarily equal the difference between the initial number of trials and the final number of trials reported.

| **Model** | **Method** | **Subevent No. Change** | **Content Accuracy Change** | **No. of Detail Change** | **Random factor: Participant** | **Random factor: Event** | **AIC** | **BIC** | **BF01** |
| --- | --- | --- | --- | --- | --- | --- | --- | --- | --- |
| 1 | LM | V |  |  |  |  | 411.214 | 424.968 |  |
| 2 | LMM | V |  |  | V | V | 365.173 | 388.097 |  |
| 3 | LMM | V | V |  | V | V | 373.864 | 401.372 | <.001 |
| 4 | LMM | V |  | V | V | V | 375.710 | 403.218 | 0.001 |
| 5 | LMM | V | V | V | V | V | 384.333 | 416.426 | <.001 |

Table S2. Comparison of results predicting duration of the whole event using different methods. Tick marks indicate the variables included in the model prediction. All linear mixed-effects model (LMM) analyses include participants and events as random factors. Model fits are evaluated using AIC and BIC. The AIC and BIC indicate better model fits for LMM analysis compared to LM analysis. The model comparison results show that the linear model with changes in gist accuracy and detail (Model 5) provided a worse fit relative to the best-fitting model based on BIC (Model 2; *BF01* < .001). *BF01* indicates the evidence in favor of the best-fitting model (Model 2) compared to Model 3,4,5.

| **Model** | **Method** | **No. of Detail Change** | **Character detail change** | **Action detail change** | **Action detail change** | **Place detail change** | **False detail change** | **Random factor: Participant** | **Random factor: Event** | **AIC** | **BIC** |
| --- | --- | --- | --- | --- | --- | --- | --- | --- | --- | --- | --- |
| 1 | LM |  |  |  |  |  |  | V | V | 1717.765 | 1739.005 |
| 2 | LMM | V |  |  |  |  |  | V | V | 1726.7 | 1753.249 |
| 3 | LMM |  | V | V | V | V | V | V | V | 1750.33 | 1798.119 |

Table S3. Comparison of Results Predicting Duration of the Subevent Using Different Methods. Tick marks indicate the variables included in the model prediction. All linear mixed-effects model (LMM) analyses include participants and events as random factors. Model fits are evaluated using AIC and BIC. The AIC and BIC values indicate model fit, with lower values suggesting better fit. Model 1 demonstrates the best fit based on AIC and BIC values, indicating that the number of detail changes is not a significant predictor for subevent duration changes across days.

|  | Subevent Number | Gist Accuracy | Number of Detail Recalled |
| --- | --- | --- | --- |
| Day 1 VIF  (Model in Fig S3) | 1.207 | 1.111 | 1.15 |
| Day 7 VIF  (Model in Fig S3) | 1.096 | 1.069 | 1.101 |
| Across-Days VIF  (Model in Fig 2) | 1.236 | 1.206 | 1.238 |

Table S4. Variance Inflation Factor (VIF) values assessing collinearity among predictors in the linear mixed-effects models. The fixed predictors were Subevent Number, Gist Accuracy, and Number of Details Recalled. VIF values are presented for Day 1 (Model in Fig. S3), Day 7 (Model in Fig. S3), and Across-Days (Model in Fig. 2) models. VIF values below 5 indicate minimal collinearity.

|  | Correlation between Subevent Number and Gist Accuracy | Correlation between Subevent Number and Number of Detail Recalled | Correlation between Gist Accuracy and Number of Detail Recalled |
| --- | --- | --- | --- |
| Day 1  (Model in Fig S3) | 0.246 | -0.304 | 0.114 |
| Day 7  (Model in Fig S3) | 0.152 | -0.226 | 0.165 |
| Across-Days  (Model in Fig 2) | 0.245 | -0.290 | 0.248 |

Table S5. Correlation coefficients assessing the relationships among predictors in the linear mixed-effects models. The fixed predictors were Subevent Number, Gist Accuracy, and Number of Details Recalled. Correlation values (Pearson’s r) are presented for Day 1 (Model in Fig. S3), Day 7 (Model in Fig. S3), and Across-Days (Model in Fig. 2) models.

| **Event** | **Content** | **Number of subevents** | **Duration of subevents** | **Platform** | **Creator ID** | **Creator Name** |
| --- | --- | --- | --- | --- | --- | --- |
| 1 | Old car restoration | 2 | 40-40 | Youtube | @therestorator7215 | The Restorator |
| 2 | Making lantan | 2 | 40-40 | Youtube | @Topaau | Topaau |
| 3 | BBQ | 2 | 40-40 | Youtube | @babishculinaryuniverse | Babish Culinary Universe |
| 4 | Making a wall clock | 2 | 40-40 | Youtube | @PouringYourHeartOut | Pouring Your Heart Out |
| 5 | Cutting vegetables | 2 | 40-40 | Youtube | @omgfollowe8684 | OMG FOLLOW E |
| 6 | Making glass vase | 2 | 40-40 | Youtube | @corningmuseumofglass | Corning Museum of Glass |
| 7 | Making a notebook | 2 | 40-40 | Bilibili | SHENG声声慢 | SHENG声声慢 |
| 8 | Playing at the beach | 2 | 40-40 | Youtube | @evelinaameliadamir2557 | Evelina Amelia Damir |
| 9 | Building bookshelf | 2 | 40-40 | Youtube | @EpicWoodworking | Epic Woodworking |
| 10 | Making diy beans | 2 | 40-40 | Bilibili | 尔沁没有科 | 尔沁没有科 |
| 11 | Army training | 3 | 20-20-40 | Youtube | @CorporalStock | Recruit Training Videos - Corporal Stock |
| 12 | Making concrete pad | 3 | 20-20-40 | Youtube | @HomeRenoVisionDIY | Home RenoVision DIY |
| 13 | Football training | 3 | 40-20-20 | Youtube | @7mlc | 7mlc |
| 14 | Japanese drawing | 3 | 20-20-40 | Youtube | @AlbertaUArts | AlbertaUArts |
| 15 | Making Tofu | 3 | 20-20-40 | Youtube | @delicacyhunter | Delicacy Hunter |
| 16 | Making Mochi | 3 | 20-20-40 | Youtube | @Terry-Films | Terry Films |
| 17 | Jackfruit cutting | 3 | 40-20-20 | Youtube | @Terry-Films | Terry Films |
| 18 | Making donut | 3 | 40-20-20 | Youtube | @Jackyt92 | Jaclynn |
| 19 | Playing at the carnival | 3 | 20-40-20 | Youtube | @WinnerEveryTime | Winner Every Time |
| 20 | Making a guitar | 3 | 20-40-20 | Youtube | @GabiM3112 | Gabriele Réti |
| 21 | Cooking lamb leg | 4 | 20-20-20-20 | Youtube | @LilRedHeidiHood | Lil Red Heidi Hood |
| 22 | Washing a car | 4 | 20-20-20-20 | Youtube | @VermijlCarDetail | Vermijl Car Detail |
| 23 | Making dumplings | 4 | 20-20-20-20 | Bilibili | @afgvillage5518 | Afg Village |
| 24 | Making shoes | 4 | 20-20-20-20 | Youtube | @TerryKimShoemaker | Terry Kim Shoemaker |
| 25 | Making paper | 4 | 20-20-20-20 | Youtube | @BestUnintentionalASMR | Best Unintentional ASMR |
| 26 | Making pottery vase | 4 | 20-20-20-20 | Youtube | @floriangadsby | Florian Gadsby |
| 27 | Installing a keyboard | 4 | 20-20-20-20 | Bilibili | 玩键盘的黄Sir | 玩键盘的黄Sir |
| 28 | Making coffee | 4 | 20-20-20-20 | Bilibili | 一棵大宝吹 | 一棵大宝吹 |
| 29 | Washing a carpet | 4 | 20-20-20-20 | Bilibili | @lubuskie_centrum_czystości | LUBUSKIE CENTRUM CZYSTOŚCI |
| 30 | Doing a haircut | 4 | 20-20-20-20 | Bilibili | @asmrmunuronkan | ASMR Münür Önkan |
|  |  |  |  |  |  |  |
| PV1 | Collecting honey | 2 | 10-26 | Youtube | @Advoko | Advoko MAKES |
| PV2 | Making bread | 4 | 7.5-22.5-7.5-7.5 | Youtube | @Stories2024 | Village lifestyle |
| PV3 | Old painting restoration | 3 | 22.5-30-22.5 | Youtube | @BaumgartnerRestoration | Baumgartner Restoration |
| PV4 | Making sandwishes | 2 | 22.5-22.5 | Youtube | @DoughertyDozen | Dougherty Dozen |

Table S6. Characteristics of video stimuli used in the experiment. Thirty primary video stimuli (Events 1-30) were systematically categorized into three conditions based on subevent quantity: 2-subevent videos (Events 1-10), 3-subevent videos (Events 11-20), and 4-subevent videos (Events 21-30). Four additional practice videos (PV1-PV4) with varied temporal structures were included for participant familiarization. Duration values indicate the temporal length of individual subevents in seconds, with hyphenated notation representing sequential subevent durations (e.g., "20-20-40" denotes three consecutive subevents of 20-, 20-, and 40-second durations, respectively). All stimuli were sourced from established video-sharing platforms, YouTube and Bilibili (See Methods for sourcing criteria). Creator ID corresponds to the platform-specific username identifier, while Creator Name represents the associated channel or content creator designation.
